# An Interaction Library for the FcεRI Signaling Network

**DOI:** 10.3389/fimmu.2014.00172

**Published:** 2014-04-15

**Authors:** Lily A. Chylek, David A. Holowka, Barbara A. Baird, William S. Hlavacek

**Affiliations:** ^1^Department of Chemistry and Chemical Biology, Cornell University, Ithaca, NY, USA; ^2^Los Alamos National Laboratory, Theoretical Division, Center for Non-linear Studies, Los Alamos, NM, USA

**Keywords:** immunoreceptor signaling, IgE receptors (FcεRI), mast cells, knowledge engineering, computational modeling, network motifs, feed-forward loops, visualization

## Abstract

Antigen receptors play a central role in adaptive immune responses. Although the molecular networks associated with these receptors have been extensively studied, we currently lack a systems-level understanding of how combinations of non-covalent interactions and post-translational modifications are regulated during signaling to impact cellular decision-making. To fill this knowledge gap, it will be necessary to formalize and piece together information about individual molecular mechanisms to form large-scale computational models of signaling networks. To this end, we have developed an interaction library for signaling by the high-affinity IgE receptor, FcεRI. The library consists of executable rules for protein–protein and protein–lipid interactions. This library extends earlier models for FcεRI signaling and introduces new interactions that have not previously been considered in a model. Thus, this interaction library is a toolkit with which existing models can be expanded and from which new models can be built. As an example, we present models of branching pathways from the adaptor protein Lat, which influence production of the phospholipid PIP_3_ at the plasma membrane and the soluble second messenger IP_3_. We find that inclusion of a positive feedback loop gives rise to a bistable switch, which may ensure robust responses to stimulation above a threshold level. In addition, the library is visualized to facilitate understanding of network circuitry and identification of network motifs.

## Introduction

Cell signaling plays a key part in regulation of the immune system. Adaptive immune responses are controlled by multi-chain immune recognition receptors, or immunoreceptors, which include the T cell receptor (TCR) (1), the B cell antigen receptor (BCR) (2), and the high-affinity receptor for IgE, which is also known as FcεRI (3). Each of these receptors is the gatekeeper of complex signaling machineries that translate extracellular stimuli into cellular responses. Individual interactions in immunoreceptor signaling systems have been studied extensively, and there is now a need to form a cohesive picture of how these interactions combine to mediate information processing. This need is driven in part by emerging data that reveal complex dynamical behaviors that arise from molecular interactions (4, 5), as well as by a growing appreciation of network features, such as crosstalk (6), which may only be apparent when one considers the interplay of multiple interactions.

Knowledge about signaling can be combined and synthesized into multiple forms, of which we employ two that are versatile and extensible: a visual map drawn in accordance with recommended standard conventions, and a rule-based model. The value of a standardized map, as opposed to an *ad hoc* cartoon, in depicting molecular interactions has been well appreciated: such maps can be used to organize information concisely, can be interpreted with minimal ambiguity, and can aid in logical analysis (7–11). After creation of a map, construction of a computational model can be viewed as the next level of information formalization (12). Through modeling, assumptions about molecular interactions (e.g., whether or not two interactions are competitive) are made more concrete and can thus be better assessed. In addition, modeling can extend our predictive capabilities when quantitative factors are important, enabling us to develop more sophisticated hypotheses. Modeling has become an increasingly important part of studies of immunoreceptor signaling (13).

Of the modeling frameworks that have been used to investigate biochemical systems, the framework of chemical kinetics is useful for studying dynamical behaviors that evolve on >1 ms time scales and that can be characterized using measurable parameters, such as protein copy numbers and binding rate constants. Among the modeling techniques of chemical kinetics is rule-based modeling (14), which provides a means to represent individual biomolecular sites, which is essential when, for example, different phosphorylation sites can recruit different binding partners (15). Rule-based modeling also enables simulation of the behavior of a large number of distinct chemical species. Myriad multicomponent protein complexes and protein phosphoforms, for example, can potentially arise in cell signaling systems and this complexity poses a challenge for other modeling techniques (16, 17). Rule-based models are built from executable rules. Rules in a model have a certain degree of interdependence, but tend to be more modular than the component parts used in other modeling techniques, such as ordinary differential equations (17). Thus, it is not only possible to formulate rules for a specific model, but to construct general rule libraries from which different models may be built.

To further our systems-level understanding of immunoreceptor signaling, we have developed a map and a rule library for early signaling mediated by FcεRI, which shares features with other related immunoreceptors. The FcεRI signaling system has a special feature of experimental tractability because the receptor can be stimulated using structurally defined antigens (18–20), making it a valuable model system for understanding how signaling is initiated. Furthermore, FcεRI has been the subject of several past modeling studies that have elucidated early events following receptor crosslinking (21, 22), the flow of information during signaling (23), aggregation of adaptor proteins (24, 25), and the impact of ligand dose and binding kinetics on kinase activation (26, 27). Aspects of the models used in these studies form a foundation for the rule library presented here. The library extends previous work by adding rules for interactions not previously included in models for FcεRI signaling. Thus, the library serves as a bridge between past studies of relatively small scope, and potential future studies that integrate information about more network elements to, for example, analyze multiplexed signaling data (28). As a first example of library use, we present simulations of recruitment of signaling proteins to the adaptor Lat, which is phosphorylated in response to FcεRI stimulation (29).

## Methods

We developed a library of rules based on known protein–protein and protein–lipid interactions, which were identified through a survey of the FcεRI literature. The rules can be assembled into different sets to form different models that capture the chemical kinetics of FcεRI signaling with site-specific resolution (14, 16, 30). Here, the term “site” is used to refer to a generic functional site in a biomolecule, which in the case of a protein may be a domain, linear motif, or amino acid residue subject to post-translational modification. In a rule-based model, rules capture knowledge about biomolecular interactions of interest. The rules in a model specify what interactions can occur in a system and under what conditions these interactions occur. A rule provides necessary and sufficient conditions for testing its applicability, a definition of the consequences of an interaction, and a rate law. A detailed example of a rule is illustrated graphically in Figure S1 in Supplementary Material. Rules, in combination with parameters and initial conditions, can be processed to simulate the time-dependent behavior of a signaling system, including the time-dependent formation of protein complexes and post-translational modifications of proteins at specific sites. A benefit of a rule-based approach is that it enables concise specification and efficient simulation of models that include multivalent interactions and multi-site phosphorylation, which are two inherent characteristics of immunoreceptor signaling systems that are otherwise difficult or impossible to fully capture in a physicochemical model. We specified our library using a domain-specific language for rule-based modeling, the BioNetGen language (BNGL) (30), which is compatible with several software tools for simulation and analysis.

Our simulations are based on the law of mass action and an assumption of well-mixed reaction compartments. In the example model, the following compartments are considered implicitly: the cytosol, the plasma membrane, and the extracellular fluid surrounding a single-cell. Simulations were performed using CVODE (31), the built-in deterministic simulator of BioNetGen, which takes as input the ODEs derived from a rule-specified reaction network. Our illustrations of rules are based on published guidelines for model visualization (10) and were drawn with the help of a template available online (http://bionetgen.org/index.php/Extended_Contact_Maps).

In our bifurcation analyses, we found stable steady states through simulations that were started from arbitrary initial conditions or nearby steady states. The bifurcation parameter was an input signal taken in the model of interest to control the rate of activation of Syk and Fyn, which were each deactivated through a first-order process. Thus, as the input signal increases, so too do the steady-state levels of active Syk and Fyn. In simulations performed to find stable steady states, the bifurcation parameter was systematically varied from a low to high value, and vice versa.

To characterize signaling dynamics for specific observables (i.e., model outputs), we calculated rise time as the time required for the observable to reach 95% of its final steady-state value. For comparison between two models, a ratio of rise times was calculated.

## Results and Discussion

### Library

In this section, we present a collection of rules, which can be viewed as a single model or as an assemblage of multiple models. Our main purpose is not to simulate the full set of interactions represented by these rules, but to formalize available knowledge about the FcεRI system to facilitate future modeling studies aimed at addressing specific questions. Rules in the library are provided in File S1 in Supplementary Material.

**Figure 1:**
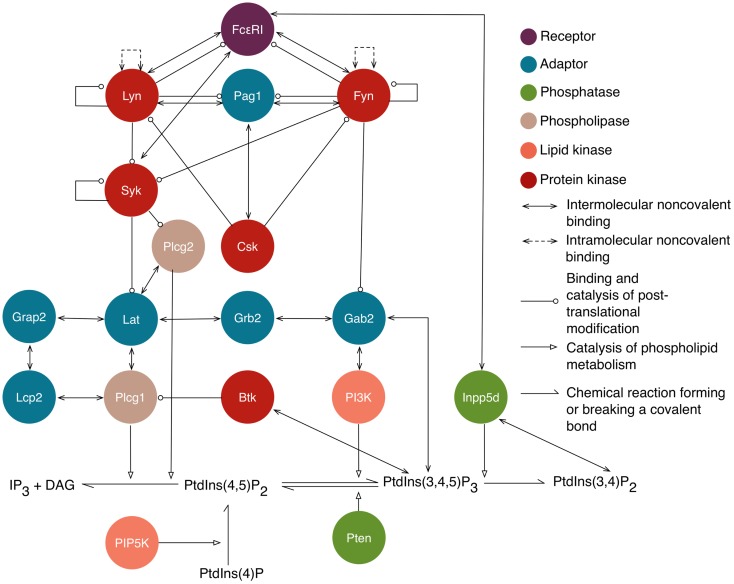
**An overview of intracellular signaling interactions included in the model/library for FcεRI signaling**. Rules are included in the library for the interactions depicted here. Proteins are represented as circles that are color-coded according to their function, as indicated in the legend. Standard UniProt names are used, and we note that Grap2 is commonly known as Gads, Lcp2 is commonly known as Slp76, and Inpp5d is commonly known as Ship1. The legend also indicates the arrows that are used to represent different types of interactions and influences. Reactions of lipid species are illustrated at the bottom. Arrows from proteins that point to lipid reactions indicate that the reaction is catalyzed by that protein. Arrows from protein to lipid species indicate that the protein binds that lipid. Not shown are implicit phosphatase reactions that cause dephosphorylation of all sites that can be phosphorylated. Ligand–receptor interactions are shown in Figure 2. A subset of interactions is illustrated with site-specific detail in Figure 3.

Rule-based models are compositional, meaning that rules can be specified somewhat independently of each other, enabling construction of new models from components of existing models. We have taken advantage of this feature to build on three previously reported models: one for ligand–receptor interactions and two for intracellular signaling. Below, we briefly review these models and the processes that they capture. A visual overview of the intracellular processes captured in the library is provided in Figure 1.

**Figure 2:**
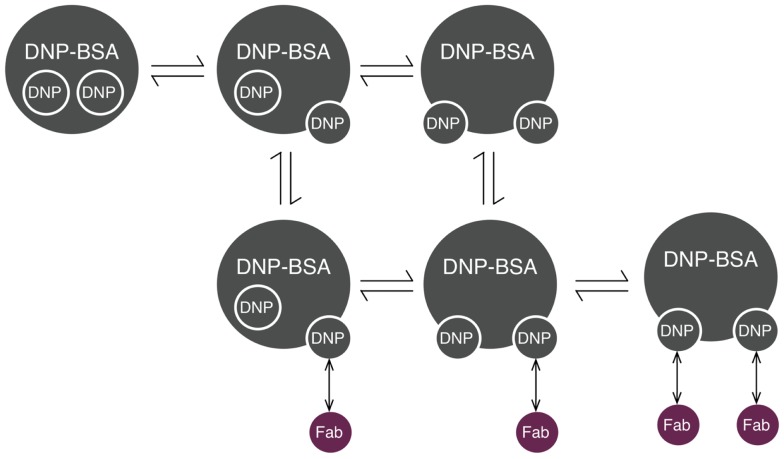
**Reaction scheme for DNP–BSA interactions with cell-surface IgE**. BSA (bovine serum albumin) is haptenated with multiple DNP groups, which are assumed to transition between two states: inaccessible (represented as being inside the molecule) and accessible (represented as being on the edge of the molecule). Accessible DNP can bind Fab arms of IgE. Each IgE antibody has two Fab arms, and is thus bivalent.

Initiation of signaling by FcεRI requires aggregation of receptors, which can be induced by reagents such as haptenated proteins and polymers, as well as by anti-receptor antibodies (32). Several models have been developed to investigate the interactions that lead to receptor aggregation. The model that we consider here is that of Xu et al. (33) for interactions of IgE-FcεRI with DNP–BSA, a multivalent antigen (haptenated protein). We chose this model because DNP–BSA is commonly used for stimulation of mast cells sensitized with anti-DNP IgE, and receptor aggregation induced by this antigen has been studied in detail (34). In this model, the effective valence of the ligand was taken to be two. The model includes transient hapten exposure, initial binding of a ligand to a receptor, crosslinking of neighboring receptors, and dissociation of ligand–receptor bonds. In this model, it was assumed that receptor sites (antigen-combining sites in cell-surface IgE) are equivalent and that the single-site dissociation rate constant is the same for both ligand sites, regardless of whether the second site is bound or free. Cyclic aggregates are not considered. For use in this study, the model was translated from its original form to rules, which was also done in another recent study (35). The model of Xu et al. is illustrated in Figure 2.

Receptor aggregation initiates signaling by bringing receptors into proximity with the Src-family kinase (SFK) Lyn. Lyn’s association with receptors may be facilitated by several complementary mechanisms, including regulation by the membrane lipid environment (36) and constitutive direct binding to FcεRI via Lyn’s unique N-terminal domain (37). For simplicity, we explicitly model the latter mechanism because it allows the plasma membrane to be treated as well-mixed and has been formalized in past modeling studies (21, 22). Lyn mediates phosphorylation of other receptors in an aggregate, thereby generating binding sites for the SH2 domain of Lyn. In this model, FcεRI constitutively associates with the unique N-terminal domain of Lyn. Crosslinking of receptors enables Lyn to *trans* phosphorylate a second receptor at sites in the receptor’s cytoplasmic subunits. These subunits, a *β* chain (Ms4a2) and a homodimer of two γ chains (Fcer1g), each contain an immunoreceptor tyrosine-based activation motif (ITAM). Each ITAM contains two (canonical) tyrosine residues that can be phosphorylated. The *β* chain contains an additional, non-canonical tyrosine in the middle of the ITAM sequence. In the original model, the tyrosines in the *β* chain were treated as a single-site, as were tyrosines in the *γ* chains. Here, we consider the *β* chain’s N-terminal (canonical) and middle (non-canonical) tyrosines separately because they are capable of recruiting distinct binding partners. The phosphorylated N-terminal tyrosine recruits Lyn to aggregated receptors via SH2 domain binding, and enhances Lyn’s catalytic activity by disruption of an inhibitory intramolecular bond, forming a positive feedback loop. The non-canonical phosphotyrosine binds the lipid phosphatase Inpp5d (Ship1), which we will discuss below. The dually phosphorylated *γ* ITAM binds the tandem SH2 domains of the kinase Syk. Tyrosine residues in the linker region of Syk are phosphorylated by Lyn. Syk *trans* phosphorylates the activation loop in a second Syk molecule that is co-localized by being bound to cross-linked receptor, which constitutes positive feedback.

Rules for additional interactions among signaling proteins, which include mediators of negative regulation, were adapted from a model for BCR signaling (38). Lyn and a second SFK, Fyn, bind the transmembrane adaptor protein Pag1. Pag1 can then be phosphorylated by these kinases, generating additional binding sites for Lyn and Fyn, as well as for the kinase Csk. When co-localized on Pag1, Csk can phosphorylate Lyn and Fyn at an inhibitory C-terminal tyrosine. In this model, it was assumed that phosphorylation occurs in *cis*, meaning that Csk mediates phosphorylation of an SFK only when both are bound to the same Pag1 molecule. The C-terminal phosphotyrosine of an SFK forms an intramolecular bond with the SFK’s SH2 domain, resulting in autoinhibition of the SFK’s kinase domain.

**Figure 3:**
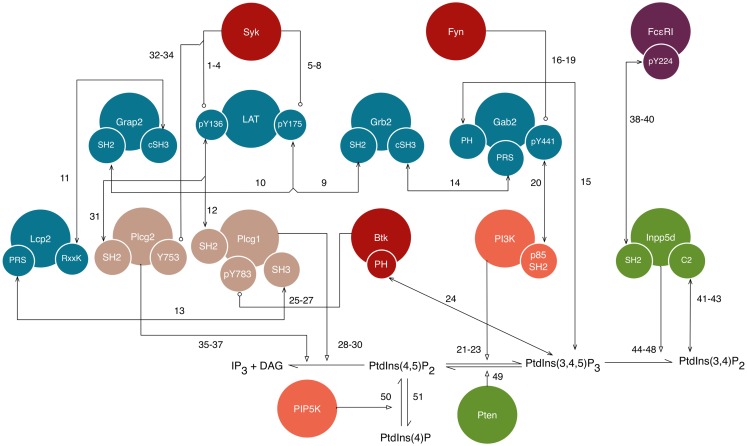
**A detailed illustration of new interactions included in the model for FcεRI signaling**. This diagram shows a subset of the interactions shown in Figure 1, but with illustration of additional details, namely the sites responsible for interactions. Conventions for color-coding and arrow symbols are the same as in Figure 1. Large circles represent proteins. Small circles, overlapping the edges of large circles, represent protein sites/components, such as domains, motifs, and amino acid residues. Standard UniProt names are used, and we note that Grap2 is commonly known as Gads, Lcp2 is commonly known as Slp76, and Inpp5d is commonly known as Ship1. Arrows represent interactions and are numbered to correspond to the numbering of rules given in the text. Phosphatase activity is considered implicitly as dephosphorylation reactions that apply to all sites, and is not illustrated in this figure.

The new rules of our library join the proximal signaling events described above to downstream processes that have not previously been considered in mechanistic models of FcεRI signaling. New rules are discussed in the sections that follow and are illustrated in Figure 3. The nomenclature and residue numbers used are consistent with UniProt conventions for rat proteins (39), because rat cells are commonly used in experimental studies of FcεRI signaling. If we view the rules of our library as constituting a single model, then the terminal output of the model is production of IP_3_, which is a second messenger. Binding of IP_3_ to its receptor on the endoplasmic reticulum leads to release of Ca^2+^ ions from intracellular stores, which is a key step for several processes in mast cell function, including degranulation and chemotaxis (40). Finally, we note that the interactions included in this library are not all unique to FcεRI signaling and are shared by pathways operative in TCR and BCR signaling. Thus, to facilitate identification of rules applicable to multiple pathways/cell types, in Table S1 in Supplementary Material, we list protein–protein interactions included in the FcεRI library and whether each interaction is part of TCR and BCR signaling according to the NetPath database (41).

### Phosphorylation of Lat

Lat is a transmembrane, palmitoylated adaptor protein (42) that is involved in many signaling processes in both T cells and mast cells (43, 44). Syk phosphorylates Lat at multiple tyrosine residues (43), of which we focus on two: Y136 and Y175, which are better known as Y132 and Y191 in human Lat. Recent imaging studies suggest that Lat and the receptor become co-clustered after antigen-mediated receptor aggregation (45, 46). However, it is not clear if Syk-mediated phosphorylation of Lat takes place within the context of a signaling complex that co-localizes Syk and Lat, or if instead, Syk-mediated phosphorylation of Lat takes place through random collisions between Syk’s kinase domain and tyrosine substrates in Lat that generate short-lived enzyme–substrate complexes, as in a Michaelis–Menten mechanism. It has previously been assumed that the latter mechanism holds (47) and we follow this approach, using rules capturing enzyme–substrate binding, dissociation, and catalysis. For example, the rules listed below, which are written using the conventions of BNGL (30), represent Syk-catalyzed phosphorylation of Y136 in Lat. Mass action kinetics are assumed. Bond indices are prefixed with the “!” symbol and internal state labels are prefixed with the “~” symbol. Here, internal state labels indicate whether a tyrosine residue is phosphorylated (“P”) or unphosphorylated (“0”).

(1) Syk(tSH2! + ,PTK) + Lat(Y136~0) - > Syk(tSH2! + ,PTK!1).Lat(Y136~0!1) kfSykLat
(2) Syk(PTK!1).Lat(Y136~0!1) - > Syk(PTK) + Lat(Y136~0) krSykLat
(3) Syk(PTK!1,Y519_Y520~P).Lat(Y136~0!1) - > Syk(PTK,Y519_Y520~P) + Lat(Y136~P) kpSykLat136_1
(4) Syk(PTK!1,Y519_Y520~0).Lat(Y136~0!1) - > Syk(PTK,Y519_Y520~0) + Lat(Y136~P) kpSykLat136_2

The first rule represents binding of Syk to Lat. In general, for rules in our library, protein components’ names are consistent with terminology used in the biological literature. Here, the PTK component of Syk represents the protein tyrosine kinase domain of the protein. We assume that the interaction represented by Rule 1 only occurs when Syk is recruited to the plasma membrane, through binding of its tandem SH2 domains (tSH2) to phosphorylated FcεRI. Thus, the rule specifies that the tSH2 component must be bound for the reaction to occur (indicated by “!+”). The second rule represents the reverse reaction, which occurs independently of the binding state of Syk. Thus, the tSH2 component of Syk is not included in this rule. Rules 3 and 4 represent phosphorylation of Lat Y136 by Syk. These two rules differ in whether Syk is phosphorylated at its activation loop tyrosine residues Y519 and Y520, which are treated as a single site for simplicity. Phosphorylation of the activation loop enhances the catalytic activity of Syk (48). Rate constants consistent with this regulatory mechanism are given after each rule, and are assigned values in the “parameters” block of the model specification (File S1 in Supplementary Material). A similar set of rules are used to capture phosphorylation of Y175 in Lat.

(5) Syk(tSH2! + ,PTK) + Lat(Y175~0) - > Syk(tSH2! + ,PTK!1).Lat(Y175~0!1) kfSykLat
(6) Syk(PTK!1).Lat(Y175~0!1) - > Syk(PTK) + Lat(Y175~0) krSykLat
(7) Syk(PTK!1,Y519_Y520~P).Lat(Y175~0!1) - > Syk(PTK,Y519_Y520~P) + Lat(Y175~P) kpSykLat175_2
(8) Syk(PTK!1,Y519_Y520~0).Lat(Y175~0!1) - > Syk(PTK,Y519_Y520~0) + Lat(Y175~P) kpSykLat175_1

### Interactions among Lat and its binding partners

Phosphorylated Y136 and Y175 have preferences for distinct binding partners, although crosstalk occurs between the pathways that branch from each site. Phosphorylated Y175 binds Grb2 and Grap2 (commonly known as Gads) (49), which are two related cytosolic adaptor proteins that each contain an SH2 domain flanked by two SH3 domains (50). These adaptors are also able to bind other sites in Lat, with Grb2 being more promiscuous (51), but for simplicity we focus on Y175. The interactions of Lat pY175 with Grb2 and Grap2, which are taken to be mutually exclusive, are modeled as follows:

(9) Lat(Y175~P) + Grb2(SH2) < - > Lat(Y175~P!1).Grb2(SH2!1) kfLatGrb2, krLatGrb2
(10) Lat(Y175~P) + Grap2(SH2) < - > Lat(Y175~P!1).Grap2(SH2!1) kfLatGrap2, krLatGrap2

These rules are nearly as general as possible, in that minimal molecular context is included on the left-hand side of either of these rules (i.e., the only requirements for a bond to form is availability of the cognate binding sites in each molecule). For this reason, a large number of distinct reactions are implicitly defined by each rule. This feature is a generic aspect of rules and what allows for concise model specification.

Grap2 binds Lcp2, which is also known as Slp76. This high-affinity interaction occurs through the SH3 domain of Grap2 and an unconventional RxxK motif in Lcp2 (52).

(11) Grap2(SH3) + Lcp2(RxxK) < - > Grap2(SH3!1).Lcp2(RxxK!1) kfGrap2Lcp, krGrap2Lcp

Phosphorylated Y136 in Lat binds phospholipase C*γ*1 (Plcg1) with high specificity (49), and the interaction is modeled with the following rule:

(12) Lat(Y136~P) + Plcg1(SH2) < - > Lat(Y136~P!1).Plcg1(SH2!1) kfLatPlcg, krLatPlcg

Both of the tandem SH2 domains of Plcg1 contribute to co-localization of this enzyme with FcεRI upon stimulation (53), and there is evidence both SH2 domains are capable of binding Lat (54). However, for simplicity, we only consider a single SH2 domain in this model.

Plcg1 also interacts with Lcp2, via the SH3 domain of Plcg1 (55).

(13) Lcp2(PRS) + Plcg1(SH3) < - > Lcp2(PRS!1). Plcg1(SH3!1) kfLcp2Plcg1,krLcp2Plcg1

The final adaptor protein that we consider is Gab2. A linear motif in Gab2 can bind to the C-terminal SH3 domain of Grb2. We designate this motif as a proline-rich sequence (PRS), although its sequence differs from conventional SH3 binding motifs (56).

(14) Grb2(cSH3) + Gab2(PRS) < - > Grb2(cSH3!1). Gab2(PRS!1) kfGrb2Gab2,krGrb2Gab2

In addition, Gab2 can be recruited by binding of its PH domain to phosphatidylinositol 3,4,5-trisphosphate [PtdIns(3,4,5)P_3_], also abbreviated as PIP_3_, in the plasma membrane (57).

(15) PI345P3(headgroup) + Gab2(PH) < - > PI345P3(headgroup!1).Gab2(PH!1) kfGab2Pip3,krGab2Pip3

The “headgroup” component in these rules represents the headgroup of the lipid, which is responsible for interactions with proteins.

### Recruitment of PI3K to Gab2

PI3K association with Gab2 is dependent on Gab2 phosphorylation. Gab2 is phosphorylated by Fyn (58), which we assume catalyzes phosphorylation through a Michaelis–Menten mechanism.

(16) Fyn(U! + ,SH2,PTK) + Lat(Y175~P!1). Grb2(SH2!1,cSH3!2).Gab2(PRS!2,Y441~0) - > Fyn(U! + ,SH2,PTK!3).Lat(Y175~P!1). Grb2(SH2!1,cSH3!2).Gab2(PRS!2,Y441~0!3) kfFynGab2
(17) Rec(b_Y210~P!4).Fyn(U,SH2!4,PTK) + Lat(Y175~P!1).Grb2(SH2!1,cSH3!2). Gab2(PRS!2,Y441~0) - > Rec(b_Y210~P!4). Fyn(U,SH2!4,PTK!3).Lat(Y175~P!1). Grb2(SH2!1,cSH3!2).Gab2(PRS!2,Y441~0!3) kfFynGab2
(18) Fyn(PTK!1).Gab2(Y441~0!1) - > Fyn(PTK) + Gab2(Y441~0) krFynGab2
(19) Fyn(PTK!1).Gab2(Y441~0!1) - > Fyn(PTK) + Gab2(Y441~P) kpFynGab2

The first two rules differ with respect to the mechanism by which Fyn is bound to a receptor. In the first rule, Fyn is taken to be bound by its unique domain (U). In the second rule, Fyn is taken to be bound by its SH2 domain.

Phosphorylated Gab2 binds the SH2 domain in the p85 subunit of PI3K (p85_SH2). Y441 of Gab2 lies in a consensus sequence for p85 binding (59).

(20) Gab2(Y441~P) + Pi3k(p85_SH2) < - > Gab2(Y441~P!1).Pi3k(p85_SH2!1) kfGab2Pi3k,krGab2Pi3k

### PI3K activity

Once recruited, PI3K phosphorylates the 3rd position in the inositol ring of phosphatidylinositol 4,5-bisphosphate [PtdIns(4,5)P_2_], also abbreviated as PIP_2_, generating PIP_3_.

(21) Lat(Y175~P!1).Grb2(SH2!1,cSH3!2). Gab2(PRS!2,Y441~P!3).Pi3k(p85_SH2!3, PI3Kc) + PI45P2(headgroup) - > Lat(Y175~P!1).Grb2(SH2!1,cSH3!2). Gab2(PRS!2,Y441~P!3).Pi3k(p85_SH2!3, PI3Kc!4).PI45P2(headgroup!4) kfPi3kPip2
(22) Pi3k(PI3Kc!1).PI45P2(headgroup!1) - > Pi3k(PI3Kc) + PI45P2(headgroup) krPi3kPip2
(23) Pi3k(PI3Kc!1).PI45P2(headgroup!1) - > Pi3k(PI3Kc) + PI345P3(headgroup) kpPi3k DeleteMolecules

In these rules, lipid phosphorylation is treated as consumption and production of different lipid species. For this reason, the BNGL keyword “DeleteMolecules” is used to indicate removal of reactant molecules (30).

### Btk-mediated activation of Plcg1

PtdIns(3,4,5)P_3_ is a binding partner for multiple proteins, including the Tec-family kinase Btk, which is involved in activating Plcg1. The PH domain of Btk mediates this interaction.

(24) Btk(PH) + PI345P3(headgroup) < - > Btk(PH!1).PI345P3(headgroup!1) kfBtkPip3,krBtkPip3

Recruited Btk can phosphorylate Plcg1 at sites that are associated with enhancement of phospholipase activity (60). In this way, pathways that branch from the two Lat phosphosites, Y136 and Y175, converge in contributing to IP_3_ production.

(25) Btk(PH! + ,PTK) + Plcg1(SH2! + ,Y783~0) - > Btk(PH! + ,PTK!1).Plcg1(SH2! + ,Y783~0!1) kfBtkPlcg
(26) Btk(PTK!1).Plcg1(Y783~0!1) - > Btk(PTK) + Plcg1(Y783~0) krBtkPlcg
(27) Btk(PTK!1).Plcg1(Y783~0!1) - > Btk(PTK) + Plcg1(Y783~P) kpBtkPlcg

### Plcg1 activity

Plcg1 cleaves PtdIns(4,5)P_2_ to generate the second messengers diacyl glycerol (DAG) and inositol 1,4,5-trisphosphate (IP_3_) (61). The cleavage reaction is taken to occur through a Michaelis–Menten mechanism:

(28) Plcg1(SH2! + ,PLC) + PI45P2(headgroup) - > Plcg1(SH2! + ,PLC!1).PI45P2(headgroup!1) kfPlcgPip2
(29) Plcg1(PLC!1).PI45P2(headgroup!1) - > Plcg1(PLC) + PI45P2(headgroup) krPlcgPip2
(30) Plcg1(PLC!1,Y783~P).PI45P2(headgroup!1) - > Plcg1(PLC,Y783~P) + IP3() + DAG() kcPlcg DeleteMolecules

### Recruitment and activity of Plcg2

In addition to Plcg1, we also include Plcg2 in the library because isoform-specific differences between these two proteins have been found in FcεRI signaling. It has been observed that phosphorylation and activation of Plcg2 is less sensitive to PI3K inhibition than Plcg1 (62). Thus, we include a mechanism by which Plcg2 is activated by Syk rather than by Btk. However, we note that other studies have found phosphorylation of Plcg2 to be reduced in the absence of Btk (63), suggesting that Btk may act on Plcg2.

(31) Lat(Y136~P) + Plcg2(SH2) < - > Lat(Y136~P!1).Plcg2(SH2!1) kfLatPlcg, krLatPlcg
(32) Syk(tSH2! + ,PTK) + Plcg2(SH2! + ,Y753~0) - > Syk(tSH2! + ,PTK!1).Plcg2(SH2! + , Y753~0!1) kfSykPlcg
(33) Syk(PTK!1).Plcg2(Y753~0!1) - > Syk(PTK) + Plcg2(Y753~0) krSykPlcg
(34) Syk(PTK!1).Plcg2(Y753~0!1) - > Syk(PTK) + Plcg2(Y753~P) kpSykPlcg
(35) Plcg2(SH2! + ,PLC) + PI45P2(headgroup) - > Plcg2(SH2! + ,PLC!1).PI45P2(headgroup!1) kfPlcgPip2
(36) Plcg2(PLC!1).PI45P2(headgroup!1) - > Plcg2(PLC) + PI45P2(headgroup) krPlcgPip2
(37) Plcg2(PLC!1,Y753~P).PI45P2(headgroup!1) - > Plcg2(PLC,Y753~P) + IP3() + DAG() kcPlcg DeleteMolecules

Rule 31 represents binding to Lat. Rules 32–34 represent phosphorylation of Plcg2 through a Michaelis–Menten mechanism. Rule 35–37 represent catalyzed hydrolysis of PIP_2_.

### Activation of Inpp5d

The final regulator of lipid signaling explicitly considered in our model is Inpp5d, also known as Ship1, a phosphatase that can be recruited to FcεRI by binding a non-canonical ITAM tyrosine in the *β* subunit of the receptor (64, 65). Although Inpp5d and Lyn both bind the *β* subunit, they have preferences for different phosphotyrosines and thus we treat these interactions as non-competitive. Inpp5d dephosphorylates the 5th position of the inositol ring of PtdIns(3,4,5)P_3_ to form PtdIns(3,4)P_2_. This product of Inpp5d activity can in turn bind the Inpp5d C2 domain (66), forming a positive feedback loop that has an overall negative impact on FcεRI-mediated degranulation. The following rules are used to model binding of Inpp5d to the receptor:

(38) Inpp5d(SH2,C2) + Rec(b_Y224~P) - > Inpp5d(SH2!1,C2).Rec(b_Y224~P!1) kfShipRec
(39) Inpp5d(IPP,C2! +) + Rec(b_Y224~P) - > Inpp5d(IPP!1,C2! +).Rec(b_Y224~P!1) 100*kfShipRec
(40) Inpp5d(SH2!1).Rec(b_Y224~P!1) - > Inpp5d(SH2) + Rec(b_Y224~P) krShipRec

In the first rule, Inpp5d is cytosolic, because its SH2 and C2 domains are both free and, in the model, these are the only domains that mediate membrane recruitment. In the second rule, Inpp5d is already membrane associated through binding of its C2 domain to PtdIns(3,4)P_2_. For this reason, receptor binding occurs more quickly (we assume a 100-fold enhancement). The third rule represents dissociation of Inpp5d from the receptor.

Binding of Inpp5d to PtdIns(3,4)P_2_ is modeled similarly, with different rules for membrane-recruited and cytosolic Inpp5d:

(41) Inpp5d(SH2,C2) + PI34P2(headgroup) - > Inpp5d(SH2,C2!1).PI34P2(headgroup!1) kfShipPip2
(42) Inpp5d(SH2! + ,C2) + PI34P2(headgroup) - > Inpp5d(SH2! + ,C2!1).PI34P2(headgroup!1) 100*kfShipPip2
(43) Inpp5d(C2!1).PI34P2(headgroup!1) - > Inpp5d(C2) + PI34P2(headgroup) krShipPip2

In the first rule, Inpp5d is cytosolic, whereas in the second rule, it is localized to the membrane through binding of its SH2 domain to the receptor. As above, a 100-fold enhancement is assumed. The third rule represents dissociation.

### Inpp5d activity

The following rules capture the catalytic activity of Inpp5d:

(44) Inpp5d(SH2! + ,C2,IPP) + PI345P3 (headgroup) - > Inpp5d(SH2! + ,C2,IPP!1). PI345P3(headgroup!1) kfShipPip3
(45) Inpp5d(SH2,C2! + ,IPP) + PI345P3 (headgroup) - > Inpp5d(SH2,C2! + ,IPP!1). PI345P3(headgroup!1) kfShipPip3
(46) Inpp5d(SH2! + ,C2! + ,IPP) + PI345P3 (headgroup) - > Inpp5d(SH2! + ,C2! + , IPP!1).PI345P3(headgroup!1) kfShipPip3
(47) Inpp5d(IPP!1).PI345P3(headgroup!1) - > Inpp5d(IPP) + PI345P3(headgroup) krShipPip3
(48) Inpp5d(IPP!1).PI345P3(headgroup!1) - > Inpp5d(IPP) + PI34P2(headgroup) kdpShipPip3 DeleteMolecules

In the rules above, “IPP” represents the catalytic domain of Inpp5d.

### Additional lipid reactions

Conversion of PtdIns(3,4,5)P_3_ to PtdIns(4,5)P_2_ by Pten is considered implicitly as a first-order reaction. Conversions between PtdIns(4,5)P_2_ and PtdIns(4)P are modeled similarly.

(49) PI345P3(headgroup) - > PI45P2(headgroup) kPten DeleteMolecules
(50) PI4P(headgroup) - > PI45P2(headgroup) kfP5 DeleteMolecules
(51) PI45P2(headgroup) - > PI4P(headgroup) krP5 DeleteMolecules

### Identification of network motifs

It has been hypothesized that relatively simple network motifs with specialized functions play important roles in cellular regulatory systems and that understanding the design principles of these motifs can help us better understand the complex systems in which they are embedded (67, 68). Network motifs, such as feedback loops, have the potential to generate and/or regulate non-linear dynamical behavior (69), which may, for example, enable precise encoding of information about a stimulus (70). We assessed the FcεRI signaling network for the presence of network motifs, and identified motifs from four classes: positive feedback loops, negative feedback loops, incoherent feed-forward loops, and coherent feed-forward loops. Several of the positive and negative feedbacks contribute to regulation of the SFKs Lyn and Fyn, as well as Syk. One positive feedback loop arises because SFKs phosphorylate tyrosine residues in FcεRI, which serve as binding sites that recruit additional Lyn and Fyn molecules. Furthermore, Lyn and Fyn can each *trans* phosphorylate their own activation loop, which enhances catalytic activity. A similar mechanism also activates the kinase Syk. Negative feedback arises because Lyn and Fyn can phosphorylate the adaptor Pag1, which recruits Csk to negatively regulate SFK activity. This set of interactions has been predicted to lead to oscillations in BCR signaling (38).

Other positive feedback loops are involved in regulating lipid metabolism. PI3K generates PIP_3_, which recruits Gab2. Gab2 can in turn recruit additional PI3K. An additional positive feedback loop regulates Inpp5d, because it is capable of binding its own product. Inpp5d is also involved in an incoherent feed-forward loop, meaning a process in which two parallel mechanisms have opposite influences on an output. Here, the output is PIP_3_. Inpp5d is recruited to FcεRI and dephosphorylates PIP_3_. Incoherence arises because FcεRI contributes to activation of PI3K, which generates PIP_3_. In this way, opposing influences are exerted on the abundance of PIP_3_ upon stimulation of FcεRI signaling. Such circuitry has been hypothesized to be involved in adaptation, the capacity of a system to respond to an input and then reset itself to a pre-stimulated state (71). Thus, PIP_3_ level may be raised and then lowered after a period of FcεRI stimulation, with Inpp5d-mediated positive feedback reinforcing negative regulation over time.

Finally, we identified a pair of coherent feed-forward loops stemming from the adaptor Lat. In a coherent feed-forward loop, two processes exert the same influence (either positive or negative) on an output. In each of the feed-forward loops of interest here, both processes in the network motif have a positive influence on Plcg1 activity. In the first feed-forward loop, Lat recruits Plcg1 via one of its phosphotyrosines. Other Lat phosphotyrosines are involved in assembly of a signaling complex that ultimately recruits PI3K. The product of PI3K, PIP_3_, binds the kinase Btk, which phosphorylates Plcg1 at an activating site. In the second feed-forward loop, Lat contributes to Plcg1 recruitment through direct binding as well as through recruitment of another adaptor, Lcp2. What function could be achieved by these (overlapping) feed-forward loops? In transcriptional regulatory networks, it has been found that feed-forward loops can act as sign-sensitive delay elements, meaning that they enable rapid responses to changes in an input in one direction, and slow responses to changes in the input in the opposite direction (72, 73). Thus, the feed-forward loops initiated by Lat may influence the timing of Plcg1 activation and deactivation after increases or decreases in, for example, upstream receptor phosphorylation.

It is worth noting that Plcg1 and PI3K act on the same substrate, PIP_2_. Thus, although PI3K can positively influence Plcg1, these two enzymes also compete with one another and could together deplete available PIP_2_, assuming both access the same lipid pool. In this way, the feed-forward loop may be self-limiting. For example, if Plcg1 causes rapid conversion of PIP_2_ to IP_3_, less PIP_2_ would be available to PI3K and as a result, less PIP_3_ would be generated and the impact of the feed-forward loop would be reduced. The strength of the feed-forward loop would also be influenced by the rate of production of PIP_2_ by specific lipid kinases and phosphatases. A caveat is that Plcg1 and PI3K may act on spatially distinct lipid pools, which PIP_2_ has been found to exist in (74). These factors are not immediately evident from examination of isolated circuitry. This example highlights the importance of considering broader context and physical parameters (e.g., concentrations and binding affinities) in assessment of network motif functionality.

### Sensitivity of phospholipid metabolism to protein tyrosine kinase activation

We next used our rule library to develop models for investigation of signaling dynamics. We focused on the adaptor protein Lat, which is known for its role as a signaling hub in both T cells and mast cells (44). This role arises in large part from its capacity to recruit multiple adaptors and enzymes that regulate lipid metabolism and production of second messengers. Most of Lat’s interactions depend on prior Lat phosphorylation, which is catalyzed primarily by Syk. However, studies of FcεRI signaling induced by structurally defined antigens have revealed that not all “downstream” events are equally dependent on Lat phosphorylation. Specifically, a panel of rigid antigens, composed of haptenated DNA sequences and differing in the distance between DNP hapten groups, was evaluated for the ability to induce phosphorylation of signaling proteins, Ca^2+^ mobilization, and degranulation. It was found that phosphorylation of FcεRI and Lat, as well as store-operated Ca^2+^ entry and degranulation, were strongly dependent on hapten spacing, with the shortest spacing examined associated with the strongest responses. In contrast, it was also found that release of Ca^2+^ from intracellular stores did not show as strong a dependence on the distance between hapten sites (19). Given that Ca^2+^ release is thought to occur as a result of activities of proteins that depend on Lat, how can this apparent uncoupling between Lat phosphorylation and Ca^2+^ mobilization be explained?

We hypothesize that compensatory mechanisms mediated by Fyn and Gab2 (58) are involved in this phenomenon. Gab2 can be phosphorylated by Fyn, and can then recruit PI3K. As discussed above, production of PIP_3_ by PI3K contributes to activation of Plcg1. A product of Plcg1 is IP_3_, which induces release of Ca^2+^ from intracellular stores. Thus, if Gab2 recruitment and activation is robust to differences in Lat phosphorylation level, then Gab2 may open an avenue by which Ca^2+^ mobilization could escape control of Lat. We used our rule library to build models to determine if Gab2 could potentially enable Ca^2+^ mobilization when Lat phosphorylation is diminished.

**Figure 4:**
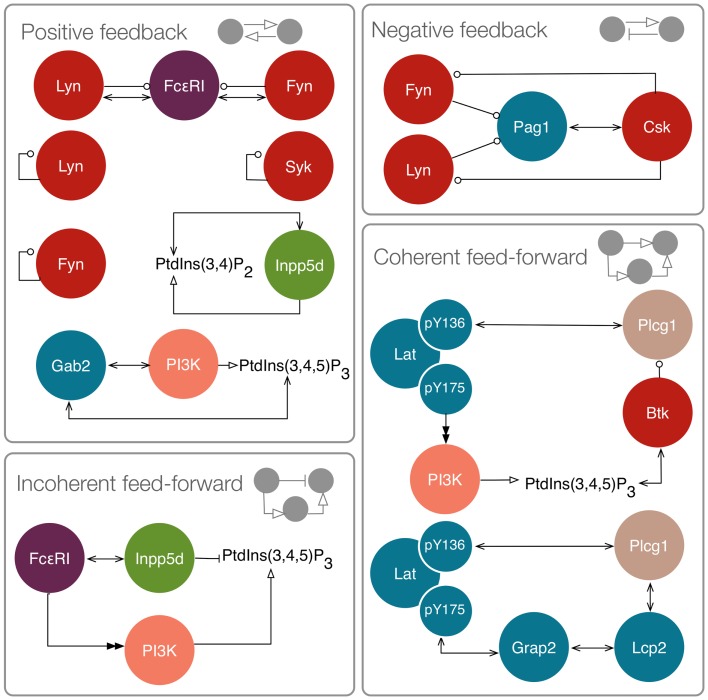
**Motifs in the FcεRI signaling network**. Positive feedbacks include interactions between FcεRI and Lyn and Fyn, because Lyn and Fyn catalyze phosphorylation of additional binding sites for these kinases. Lyn, Fyn, and Syk are subject to *trans* autophosphorylation at activating sites. Inpp5d binds its own product. Gab2 recruits PI3K, which generates PIP_3_, which can recruit additional Gab2. Negative feedback includes inhibition of Lyn and Fyn by Csk. Incoherent feed-forward includes FcεRI stimulation leading to activation of both PI3K and Inpp5d, which exert opposing influences on PIP_3_ level. Coherent feed-forwards include recruitment and activation of Plcg1, and recruitment of Plcg1 through two pathways.

**Figure 5:**
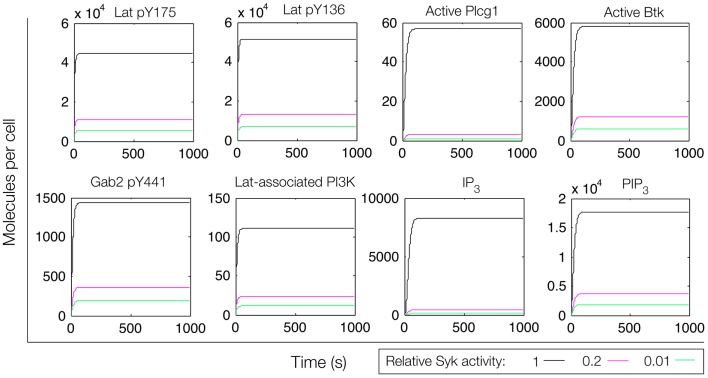
**Simulation of a model of the feed-forward loop connecting Lat to IP_3_ production**. Different color lines indicate different relative levels of Syk activity. In these simulations, Syk activity was set at the indicated level and held constant.

**Figure 6:**
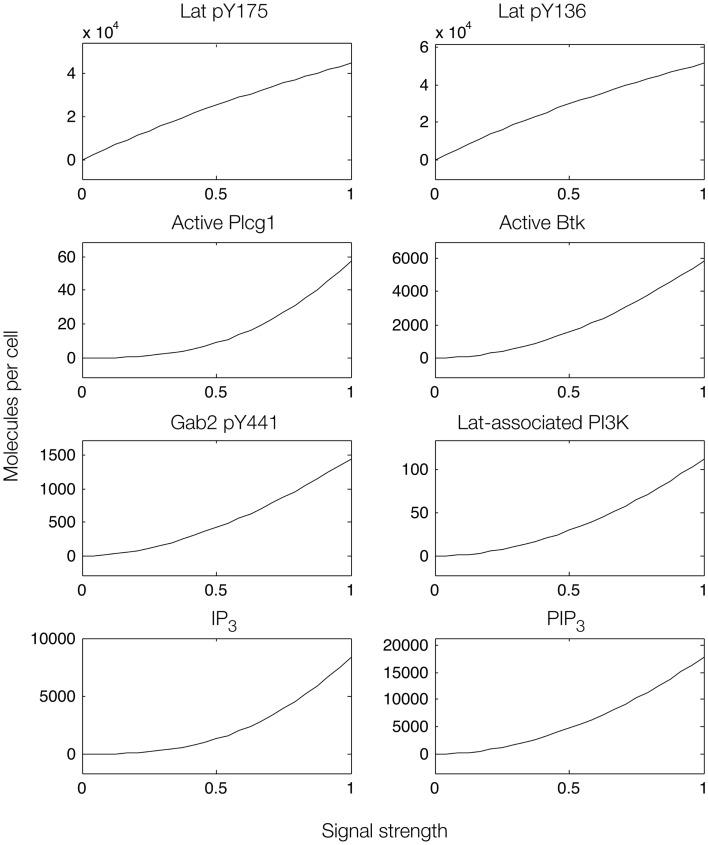
**Steady-state dose–response curves, which were found by simulation of the feed-forward loop connecting Lat to IP_3_ production**. The differences in phosphorylation level between the two phosphorylation sites in Lat (top panels) results from different affinities of the binding partners that interact with each site. Active Plcg1 is taken to be Plcg1 that is both recruited to Lat and phosphorylated, and active Btk is taken to be Btk recruited to PIP_3_.

We first considered a model in which Syk and Fyn were independent inputs. Our initial model (File S2 in Supplementary Material) essentially consists of the first coherent feed-forward loop shown in Figure 4: lat recruits Plcg1, as well as PI3K through Gab2 and Grb2. Btk is recruited to PIP_3_ and activates Plcg1 through phosphorylation. Fyn participates by phosphorylating Gab2. To model the differences between antigens observed to induce the most and least Lat phosphorylation, we considered different levels of active Syk consistent with the approximately fourfold difference in Lat phosphorylation observed experimentally (19). The level of active Fyn was kept constant. Simulations of this model revealed that differences in Lat phosphorylation level were maintained or amplified in downstream events. According to the model, a decrease in Lat phosphorylation (arising from lowered Syk activity) causes at least proportionate decreases in the levels of activated Plcg1, activated Btk, Lat-associated PI3K, PIP_3_, and IP_3_ (Figure 5). We also considered a scenario in which activity of Syk and Fyn are both controlled by the magnitude of an input signal, which may be a more realistic scenario because both kinases are recruited to phosphorylated receptors. We varied the strength of this signal and evaluated the resulting steady-state levels of outputs (Figure 6). Consistent with results from the first scenario, decreased signal strength led to decreased Lat phosphorylation, and was accompanied by even steeper decreases in activation of other signaling molecules. Thus, the interactions included in this model are insufficient to explain the experimental observation of Ca^2+^ mobilization in the absence of strong Lat phosphorylation.

**Figure 7:**
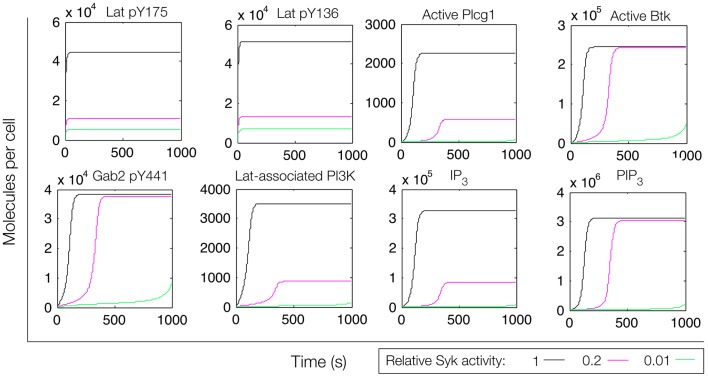
**Simulation of a model of the feed-forward loop connecting Lat to IP_3_ production with consideration of a Gab2-mediated positive feedback loop**. Different color lines indicate different relative levels of Syk activity.

In an extension of the initial model (File S3 in Supplementary Material), we incorporated additional interactions from the rule library, those responsible for the positive feedback involving Gab2 interaction with PIP_3_ (see Figure 4). We reasoned that, with the addition of these interactions, once PIP_3_ production is initiated, PIP_3_ production may become self-sustaining, because PIP_3_ is able to recruit Gab2 to the plasma membrane, which in turn is able to recruit PI3K. Simulated time courses with the same level of active Fyn and different levels of active Syk, as in Figure 5, are shown in Figure 7. These results indicate that certain signaling readouts downstream of Lat are buffered against reduced Lat phosphorylation. For example, there is less than a fourfold difference in peak IP_3_ levels between the conditions of high (black line) and intermediate (magenta line) Lat phosphorylation. In contrast, the model without Gab2-mediated positive feedback predicted a greater than 100-fold difference.

**Figure 8:**
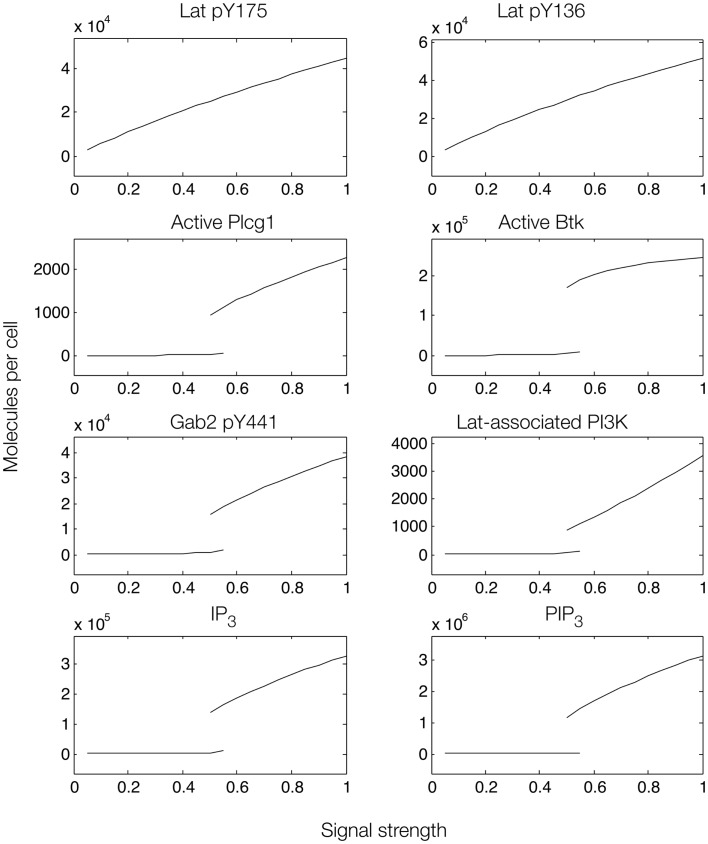
**Steady-state dose–response curves, which were found by simulation of the feed-forward loop connecting Lat to IP_3_ production when Gab2-mediated positive feedback is considered**. Each plot is a bifurcation diagram; the bifurcation parameter is signal strength, which governs the rate of production of active Syk and Fyn. Only stable steady states are shown. As can be seen, the model predicts the possibility of bistability.

To further investigate the role of positive feedback, we modulated an input signal controlling both Fyn and Syk activity, as in Figure 6. Steady-state simulation results from this model are shown in Figure 8, which differ from those obtained with the first model. First, the total numbers of signaling molecules in activated forms are greater than for the case without feedback, as long as the signal strength is above a certain level. Second, within certain input ranges, the model shows bistability, i.e., existence of two stable steady states, as indicated by signal strength values that correspond to more than one steady-state output value. Bistability has also been characterized in TCR signaling (75, 76) and BCR signaling (38, 77). Third, we found that certain signaling readouts downstream of Lat are now buffered against reduced Lat phosphorylation (Figure 7), decreasing less sharply when signal is reduced. Together, these results suggest that Gab2-mediated positive feedback may enable committed, all-or-none decisions that lead to high levels of IP_3_ as long as Lat phosphorylation is above a threshold. When input level falls below this threshold, positive feedback is unable to enhance IP_3_ production (Figure 7). Thus, some amount of PIP_3_ must be generated through Lat-dependent mechanisms before the Fyn/Gab2 pathway can contribute to production of IP_3_.

**Figure 9:**
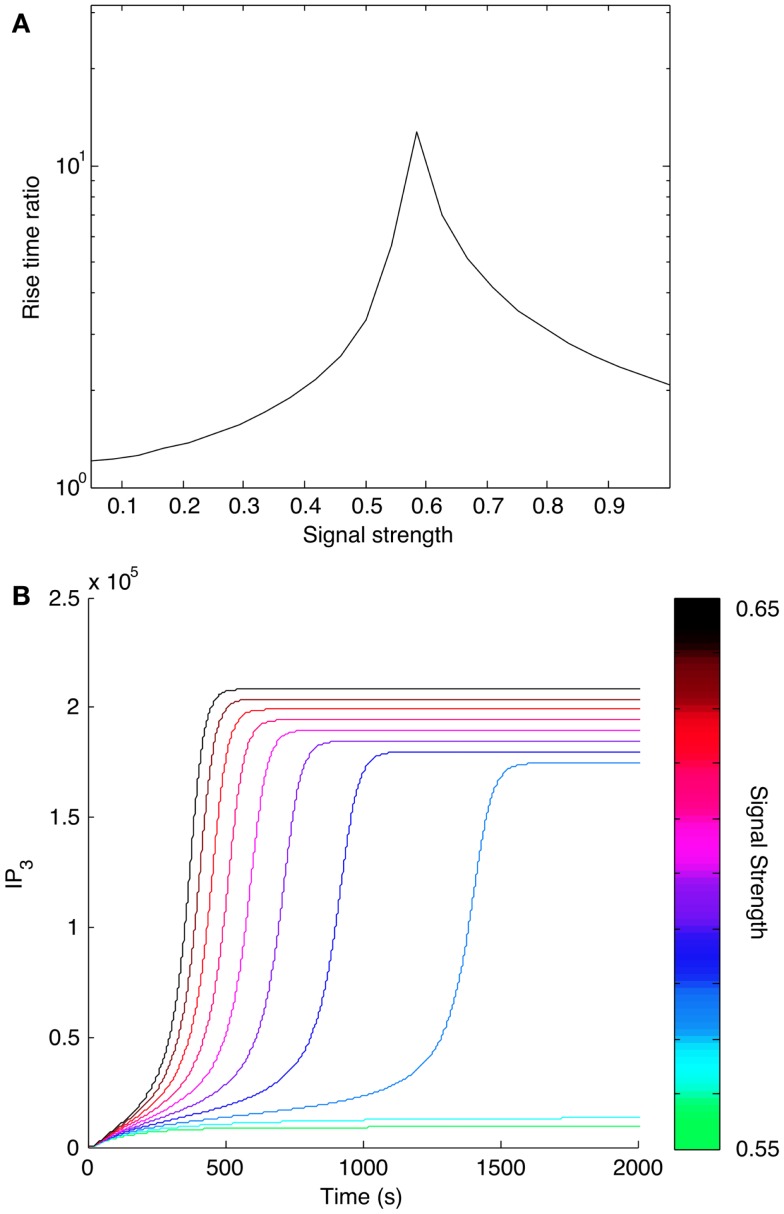
**Effect of positive feedback on signaling dynamics**. **(A)** Rise time for IP_3_ synthesis was calculated as the time needed to reach 95% of the final steady-state level. Rise times were calculated for different levels of input, or signal strength. Rise times for the model with positive feedback were divided by rise times for the model without positive feedback and plotted against corresponding input level. All indicated rise time ratios are greater than one, meaning that the model with positive feedback takes more time to reach its final steady state. **(B)** Time courses for IP_3_ production in a narrow range of input levels surrounding the peak shown in **(A)**. The input level corresponding to each curve is indicated with the color bar at the right.

We also considered how positive feedback affects the dynamics of signaling. We calculated the rise time for IP_3_ at different input levels as predicted by the models with and without positive feedback. We found that positive feedback caused IP_3_ level to reach its steady state more slowly (Figure 9A). Rise time for the model with positive feedback peaked in the bistable region, where the system transitions from a low steady state to a higher steady state (Figure 9B). The slower rise in IP_3_ level qualitatively mimics the experimentally observed dynamics of Ca^2+^ release from stores caused by antigens that induce low levels of Lat phosphorylation (19). These same antigens induce minimal store-operated calcium entry (SOCE) and minimal degranulation, which suggests that SOCE may be sensitive to the kinetics of IP_3_ production.

There are several experimental tests that could be pursued to evaluate the role of Gab2-mediated positive feedback. One predicted effect of the feedback loop is bistability of several signaling readouts (Figure 8), including PIP_3_. Testing for bistability usually benefits from single-cell measurements, because cell-to-cell variability may result in different cells having different bifurcation points. At the single-cell level, PIP_3_ production can be monitored using PH domain constructs (78). When the strength of an input signal, such as ligand-induced receptor aggregation, crosses a threshold level, the quantity of PIP_3_ is expected to increase dramatically in a switch-like manner. Another characteristic arising from bistability is hysteresis, meaning history dependence. As signal strength is reduced from a high level [e.g., by breaking up receptor aggregates with a monovalent hapten (34)], PIP_3_ level is expected to switch back to a low state. However, this switch is predicted to occur at a lower input level than that required to induce a transition from low signaling to high signaling. Controlling input level would require an understanding of how ligand dose relates to receptor aggregation, which can be obtained with a model for ligand–receptor interactions (79).

A second approach would involve disruption of the Gab2 feedback loop, which would be expected to increase sensitivity to Lat phosphorylation. Mutation of the Gab2 PH domain, which binds PIP_3_ and is therefore a key component of the feedback, would be expected to inhibit Ca^2+^ mobilization. However, such manipulation of endogenous Gab2 would be technically challenging, making this strategy potentially difficult to implement. An alternative approach would be to knock down either Gab2 or Fyn, which would be predicted to similarly inhibit Ca^2+^ mobilization.

## Conclusion

As a step toward systems-level understanding of FcεRI signaling, we have synthesized information about a relatively large number of interactions and proteins into a formalized interaction library. This library consists of executable rules that can be used to extend existing models and to build new models. The rules are annotated with information from the primary literature, thereby facilitating reuse of information. The rules are also visualized to illustrate the scope and detail of the library’s contents. Analysis of the library reveals multiple feedback and feed-forward loops in FcεRI signaling, the behavior of which can be investigated quantitatively through simulation and complementary quantitative experiments. We used the library to model events involved in phosphoinositide metabolism at different levels of Syk activity, and found that a Gab2-mediated positive feedback can compensate for reduced Lat phosphorylation, which provides a potential explanation for how antigens that induce dramatically different levels of Lat phosphorylation can induce similar Ca^2+^ fluxes (19).

We anticipate that the approach presented here will have several potential applications in linking computational and experimental investigations of cellular information processing. First, a library of rules could be used to build a model of broad scope and site-specific detail for use in analysis of multiplexed, high-resolution data, such as proteomic measurements of site-specific post-translational modifications (80). Currently, such data are often analyzed using clustering, enrichment analysis, and other techniques that reveal trends in dynamics and functions of detected proteins (81), but that do not necessarily provide a concrete picture of the mechanisms at work. Modeling will enable us to better leverage information about mechanisms and physical parameters, complementing current analysis techniques. A combination of modeling and quantitative high-throughput experimentation could, for example, be used to characterize the distribution of signaling complexes that can be nucleated by Lat. Binding partners of Lat have shared binding sites and a range of affinities (49). To understand how binding of these proteins is balanced, it would be necessary to measure binding affinities of SH2 domains to each phosphosite (82) and to quantify protein copy numbers (83). A model could then be used to integrate such data and determine the expected distribution of signaling complexes.

Second, rule libraries could facilitate the extension of models by increments. The benefit of such an approach is that a model of an idealized network motif (71, 84) could be extended piece by piece to form a more complete representation of the motif’s context. Studies of such models could reveal how well the predicted behavior of an isolated motif is maintained when additional interactions are considered, and what complicating factors may need to be taken into account in experimental assessments of motif function or in synthetic biology efforts aimed at engineering regulatory systems on the basis of network motif design principles.

Finally, rule libraries may help address problems in knowledge engineering, i.e., the task of gathering, organizing, and interpreting large quantities of information. Rule-based models have already been annotated using interactive wikis (85, 86), which could open the door to community-based model development and curation, making it easier to assemble and assess data for model building. Furthermore, a widely used approach in knowledge engineering is natural language processing (NLP), the automated derivation of information from text. A major bioinformatics goal of NLP is to extract networks and quantitative models from the primary biomedical literature (87). A limiting factor in this task is the availability of “gold standard” networks against which to compare an automatically constructed network or model, which is necessary to assess the performance of network/model construction algorithms. For many biological systems, reliable network representations and models are non-existent. Furthermore, even when a reliable network is available, it may be in a format (e.g., ordinary differential equations) that does not map to underlying interactions in a clear manner. This problem is addressed by a rule library, because rules not only serve as the basis for simulations but also provide precise, human- and machine-readable representations of biomolecular interactions. NLP could aid in library construction through automatic extraction of rules from the literature. As information about cell signaling systems continues to expand, we anticipate that formalization and synthesis of knowledge will become increasingly important for informing hypotheses, making quantitative predictions, and elucidating systems-level properties of cellular regulatory systems.

## Conflict of Interest Statement

The authors declare that the research was conducted in the absence of any commercial or financial relationships that could be construed as a potential conflict of interest.

## Supplementary Material

The Supplementary Material for this article can be found online at http://www.frontiersin.org/Journal/10.3389/fimmu.2014.00172/abstract
