# Supplementary material for: An Interaction Library for the FcεRI Signaling Network
Source: Front Immunol. 2014 Apr 15;5:172. doi: 10.3389/fimmu.2014.00172 (PMC3995055; doi:10.3389/fimmu.2014.00172)

Supplementary Figure 1

A graphical representation of how information about a biomolecular interaction (top row) is translated into a BioNetGen Language (BNGL) encoding (middle row) and a graphical representation (bottom row).

Description: Phosphorylated Y136 in Lat binds the SH2 domain in Plcg1

BNGL Encoding: `Lat(Y136~P) + Plcg1(SH2) -> Lat(Y136~P!1).Plcg1(SH2!1) kp1`

Graphical Representation:

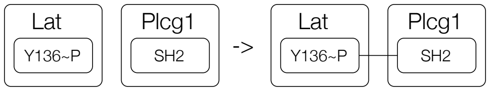

Supplement: Supplementary file 1 [file Data_Sheet_1.ZIP › ChylekLA_SupplementaryFiles/ChylekLA_SupplementaryFigure1.pdf]
