# Supplementary material for: An Interaction Library for the FcεRI Signaling Network
Source: Front Immunol. 2014 Apr 15;5:172. doi: 10.3389/fimmu.2014.00172 (PMC3995055; doi:10.3389/fimmu.2014.00172)
Supplement: Supplementary file 1 [file Data_Sheet_1.ZIP › ChylekLA_SupplementaryFiles/ChylekLA_SupplementaryTable1.pdf]

Supplementary Table 1

Shared components between FcϵRI signaling and other immunoreceptor signaling systems. Protein-protein interactions were classified as being involved (Y) or not involved (N) in BCR and TCR signaling on the basis of interactions listed for each system in the NetPath database (http://www.netpath.org/). We use NetPath as an external standard. We note that "N" merely indicates that the interaction is not currently included in NetPath. This designation does not exclude the possibility of the interaction being part of BCR or TCR signaling.

FcϵRI	BCR	TCR
FcϵRI binds Lyn	N	N
FcϵRI binds Fyn	N	N
FcϵRI binds Syk	N	N
FcϵRI binds Inpp5d	N	N
Lyn phosphorylates Lyn	N	N
Fyn phosphorylates Fyn	N	N
Syk phosphorylates Syk	Y	N
Fyn binds Pag1	N	Y
Lyn binds Pag1	N	N
Fyn phosphorylates Pag1	N	N
Lyn phosphorylates Pag1	N	N
Pag1 binds Csk	N	N
Csk phosphorylates Lyn	N	N
Csk phosphorylates Fyn	N	N
Syk phosphorylates Lat	N	N
Grb2 binds Lat	N	Y
Grap2 binds Lat	N	Y
Grap2 binds Lcp2	N	N
Plcg1 binds Lat	N	Y
Plcg1 binds Lcp2	Y	Y
Gab2 binds Grb2	N	N
Fyn phosphorylates Gab2	N	N
Gab2 binds PI3K	Y	N
Btk phosphorylates Plcg1	Y	N
Syk phosphorylates Plcg2	Y	N
